# Revealing the Correlation Between NLRP3 Inflammasome‐Related Genes and Intervertebral Disc Degeneration Based Multiomic Analysis and Experimental Validation

**DOI:** 10.1155/mi/1534745

**Published:** 2026-07-14

**Authors:** Yongliang Fu, Xiangyu Wang, Guangwei Sun, Huishuang Zou, Xianfa Du, Jinhong Fan, Xinao Li, Zhenye Yan, Min Wang, Zhen Jing, Runtian Jiang, Pingping Zhang, Bin Li

**Affiliations:** ^1^ Department of Orthopedics, First Hospital of Shanxi Medical University, Taiyuan, Shanxi, China, sxmu.edu.cn; ^2^ Department of Pain Medicine, First Medical Center, PLA General Hospital, Beijing, China, 301hospital.com.cn; ^3^ Department of Orthopedics, Shanxi Medical College Seventh Affiliated Hospital: Linfen People’s Hospital, Linfen, Shanxi, China; ^4^ Department of Orthopedics, The Second Qilu Hospital of Shandong University, Jinan, Shandong, China; ^5^ Tianjin Key Laboratory of Lung Cancer Metastasis and Tumor Microenvironment, Tianjin Medical University General Hospital, Tianjin, China, tjmugh.com.cn; ^6^ Linfen Vocational and Technical College, Linfen, Shanxi, China

**Keywords:** apoptosis, intervertebral disc degeneration, NLRP3 inflammasome, OIP5, WGCNA

## Abstract

**Purpose:**

NLRP3 inflammasome plays a pivotal role in the pathogenesis of intervertebral disc (IVD) degeneration (IDD). This study aimed to identify NLRP3 inflammasome‐related biomarkers in IDD and analyze their functions.

**Methods:**

First, the differentially expressed genes (DEGs) (IDD vs. control) in GSE124272 were detected. The NLRP3 inflammasome‐related genes (NIRGs) scores for all samples were calculated using single‐sample gene set enrichment analysis (ssGSEA). Subsequently, the key module with the strongest correlation with NIRGs scores was selected using weighted gene coexpression network analysis (WGCNA). Biomarkers were defined as the DEGs and genes showing consistent expression trends across both the GSE124272 and GSE150408 datasets. Function enrichment and immune infiltration were analyzed. The abundance of the key biomarkers was validated by real‐time quantitative PCR (RT‐qPCR). To explore the effect of core targets on IDD by constructing IDD in vitro model.

**Results:**

About 1608 intersection genes were derived from overlapping the above 2123 DEGs and 10695 key module genes. Subsequently, the top 10 genes were identified. Based on gene expression analysis, disheveled, EGL‐10, and pleckstrin domain‐containing 1 (DEPDC1), and Opa interacting protein 5 (OIP5) were selected as biomarkers. Functional enrichment analysis revealed that these biomarkers were primarily enriched in cytosolic ribosomes and DNA replication. Immune infiltration analysis identified 13 differential immune cells. Endothelial cells were inversely correlated with DEPDC1, whereas conventional dendritic cells (CDC) demonstrated a pronounced positive relationship with OIP5. RT‐qPCR results confirmed that the expression of OIP5 and DEPDC1 in IDD patients was downregulated by more than 50% (*p* < 0.05). Cell experiments showed that up‐regulation of OIP5 led to a 2‐fold reduction in cell proliferation (*p* < 0.001) and a three‐fold increase in apoptosis in nucleus pulposus (NP) cells derived from IDD patients (*p* < 0.001).

**Conclusion:**

Taken together, OIP5 has been identified as a peripheral blood biomarker linked to the NLRP3 inflammasome and may be involved in the pathogenesis of IDD.

## 1. Introduction

As a common disease, low back pain (LBP) is already a problem of global proportions [1]. Currently, intervertebral disc (IVD) degeneration (IDD), a complex and multifactorial process, is widely considered the main cause of LBP [2]. The IVD, which is composed of the central nucleus pulposus (NP), is prone to degeneration as it absorbs and distributes pressure from different directions [3]. Although medication and physical therapy can alleviate LBP in IDD patients, they cannot fundamentally reverse the degenerative process; therefore, the clinical treatment outcomes for IDD remain suboptimal [4]. Therefore, a thorough investigation into the pathophysiological mechanisms of IDD and the search for potential therapeutic targets are essential.

Inflammation is closely associated with extracellular matrix (ECM) degradation and NP cell survival, which is closely related to IDD [5, 6]. Inflammasomes are a component of innate immunity [7, 8]. NLRP3, a nucleotide‐binding oligomerization domain‐like receptor pyrin domain‐containing protein, binds to the adaptor protein ASC (apoptosis‐associated speck‐like protein containing a C‐terminal caspase recruitment domain). The assembly of these two components leads to the formation of the NLRP3 inflammasome, a complex that responds to diverse inflammation‐related stimuli [9, 10]. It is worth noting that in other inflammatory diseases, the concept of precise anti‐inflammatory management has received more and more attention. In these diseases, optimizing targets, dose windows, and delivery efficiency are considered essential for maximizing therapeutic effects while minimizing off‐target effects. For example, a recent review of dry powder inhalers highlights how organ‐targeted drug delivery can more accurately control the inflammatory pathways of lung diseases [10, 11]. NLRP3 inflammasome is associated with the development of inflammatory diseases such as rheumatoid arthritis and IDD [12, 13]. Especially, the activation of the NLRP3 inflammasome can promote the production of IL‐1β and IL‐18, further participating in the pathogenesis of IDD [14]. Therefore, a deeper understanding of the mechanisms underlying NLRP3 inflammasome may provide new insights for IDD and may also inform the development of more precise, mechanism‐based anti‐inflammatory interventions tailored to the unique disc microenvironment.

This study explored differentially expressed genes (DEGs) in IDD by means of transcriptome analysis of peripheral blood. NLRP3 inflammasome‐related genes (NIRGs) scores were calculated for all samples, and key module genes were identified using weighted gene coexpression network analysis (WGCNA). Subsequently, hub genes were screened, and those showing significant expression differences between IDD and control groups were selected as biomarkers. Based on biomarkers, gene set enrichment analysis (GSEA) enrichment analysis and immune infiltration analysis were performed. Finally, the expression and functional effects of the key biomarkers were validated in IDD patient samples and in vitro cell experiments. Although the discovery cohorts were derived from whole blood rather than local disc tissue, our findings provide a systemic perspective on IDD pathogenesis and offer experimental support for the potential of Opa interacting protein 5 (OIP5) and disheveled, EGL‐10, and pleckstrin domain‐containing 1 (DEPDC1) as peripheral blood biomarkers. These data contribute to a theoretical basis for understanding the systemic immune components involved in IDD.

## 2. Material and Methods

### 2.1. Data Extraction

The IDD‐related datasets (https://www.ncbi.nlm.nih.gov/gds) including GSE124272 and GSE150408, were utilized for our study. Specifically, GSE124272, consisting of eight IDD and eight control whole blood tissue samples, was collected based on the GPL10558 platform. Similarly, GSE150408, comprising 17 IDD patients and 17 control whole blood tissue samples, was also obtained based on the GPL10558 platform. Additionally, we identified 30 NIRGs by searching the Gene Ontology (GO) (http://geneontology.org/) and Reactome databases (https://reactome.org/). The GO IDs associated with these genes were GO:0044546 and GO:0072559, while the Reactome ID was R‐HSA‐84456.

### 2.2. Genes With Differential Expression in IDD and Control

In GSE124272, the DEGs (IDD vs. control) were identified using the “limma” package (v 3.52.4) with the threshold of *p* < 0.05, and |log_2_ fold change (FC) | > 0.5. The results were visualized using “ggplot2” (v 3.3.6) and “Complex Heatmap” (v 2.14.0) [15, 16].

### 2.3. WGCNA

The Wilcoxon test served to select the differentially expressed NIRGs (DE‐NIRGs). Subsequently, the DE‐NIRGs were compiled into a GMT gene set to calculate NIRGs scores in the GSE124272 dataset using single‐sample GSEA (ssGSEA) (“GSVA,” v 1.44.5) [17]. Analysis of the difference in NIRGs scores between IDD and control samples was conducted using the Wilcoxon test. Additionally, the key module most relevant to the NIRGs scores was identified using WGCNA (v 1.72–1). First, sample clustering was performed to detect and remove outliers. We selected a soft‐thresholding power (*β*) according to the scale‐free topology criterion. The power *β* = 8 was identified as the minimum value satisfying both a scale‐free fit index (*R* ^2^) of 0.8 and near‐zero mean connectivity. An adjacency matrix was then constructed and transformed into a topological overlap matrix (TOM). Hierarchical clustering was performed using average linkage; gene modules were then derived by dynamic tree cutting, where the minimum module size was set to 100 genes and a merging threshold for highly similar modules was set at a cut height of 0.25. Finally, modules showing the strongest positive and negative correlations with NIRGs scores were chosen for subsequent analysis.

### 2.4. Function Analysis of Intersection Genes and the Hub Genes Were Obtained

The function of intersection genes that overlap the DEGs and key module genes was explored by GO and Kyoto Encyclopedia of Genes and Genomes (KEGG) analyses (“clusterProfiler,” v 3.18.1) (adj. *p* < 0.05) and displayed (“GOplot” and “ggplot2,” v 1.0.2) [18]. The protein interaction of the intersection genes was performed using the STRING database (https://string-db.org/, confidence threshold >0.7).

To further identify hub genes, a protein–protein interaction (PPI) network was built with the STRING database (v11.5) with a combined score threshold of ≥0.4 to retain interactions with medium confidence or higher. The interaction data were imported into Cytoscape (v3.9.1), and the molecular complex detection (MCODE) plugin was utilized under default parameters (degree cutoff = 2, node score cutoff = 0.2, *k*‐core = 2, and max depth = 100) to screen the highest‐scoring cluster and extract hub genes [19]. The “RCircos” package (v 1.2.0) was utilized for the visualization of chromosomal locations of the identified hub genes. This allowed us to visualize the chromosomal locations of the identified hub genes, providing insights into their genomic distribution.

### 2.5. Biomarkers Identification

Based on the hub genes, the random forest analysis was performed with the “randomForest” (v 4.7–1.1) using the following parameters: seed = 234 and ntree = 1000. The top 10 genes ranked by mean decrease Gini were selected as candidate genes. The DEGs (*p* < 0.05) in GSE124272 and GSE150408 datasets that also exhibited consistent expression trends across both datasets were considered biomarkers.

### 2.6. Establishment and Validation of Nomogram

Construction of a nomogram based on the biomarkers was performed using the “rms” package (v 6.7–0) [20]. We then generated a calibration curve and a decision curve analysis (DCA) curve. Specifically, in the calibration curve, the model fitting index was assessed by the Hosmer–Lemeshow (HL) test, *p* > 0.05 indicated a passing result for the HL test, signifying good model performance.

### 2.7. GSEA

In GSE124272, Spearman analysis utilizing “c2.kegg.symbols.gmt” and “c5.go.symbols.gmt” from the molecular signatures database (MSigDB) (http://www.broadinstitute.org/gsea/msigdb/index.jsp) was conducted to obtain the correlation coefficients for each biomarker with all other genes, which were subsequently ordered from largest to smallest. Then, GSEA was performed through “ClusterProfiler” (adj *p* < 0.05) [21].

### 2.8. Immune Infiltration Analysis

The infiltration scores of immune cells in the GSE124272 dataset were estimated via the xCELL method (v 1.1.0) with default parameters based on the normalized gene expression matrix. Following the elimination of immune cells showing no expression in any sample, a total of 51 immune cell types were retained for subsequent analyses. The Wilcoxon test was used to assess the differences in immune cell infiltration between IDD and control samples. Spearman correlation analysis was utilized to examine the relationships among these immune cells and between the identified biomarkers and immune cells, where significance was defined as |*r*| > 0.3 and *p* < 0.05. Notably, the GSE124272 dataset was generated on a single platform (Affymetrix Human Genome U133 Plus 2.0 Array) and had undergone standard normalization (RMA) and quality control by the original contributors Biomarkers‐RNA binding protein (RBP) network and drug prediction [22].

To detect the proteins bound to the biomarkers during disease development, the Starbase database (v2.0, http://starbase.sysu.edu.cn/) was used to predict the proteins bound to the biomarkers, the screening condition was clusterNum >3, and the results were presented in the biomarkers‐RBP network via Cytoscape. Besides, to further validate the role of the biomarkers, we turned to the GeneCards (https://www.genecards.org) for the selection of proteins that are more involved in the expression and regulation of biomarkers based on the related information of biomarkers. Meanwhile, the expression of selected proteins was assessed, and their three‐dimensional constructs were downloaded from the Protein Data Bank (PDB) and AlphaFold, respectively.

### 2.9. Collection and Treatment of Clinical Specimens

A total of 40 tissue samples were harvested for this study: 20 IDD specimens from patients undergoing surgery at Linfen People’s Hospital (Qingdao) and 20 normal tissue specimens from trauma patients at Yantai Yantaishan Hospital were collected between May 2024 and January 2025. The diagnosis of IDD was established based on preoperative magnetic resonance imaging (MRI). All patients were classified as Pfirrmann III–IV. The NP tissue was obtained from 20 patients who underwent surgery for acute traumatic fractures without a history of low‐back pain or disc degeneration. Their MRI results confirmed Pfirrmann grade I–II, indicating that the IVD did not degenerate. The age distribution of IDD patients was 35–60 years (mean: 48.5 ± 7.2 years), compared with 32–58 years (mean: 46.8 ± 6.9 years) for the control group. No statistically significant difference in age was observed between the two groups. All participants signed an informed consent form (Number E20231128083) and complied with the Declaration of Helsinki.

### 2.10. Cell Culture and Transfection

Six NP samples were derived from IDD patients, and the NP cells were isolated and cultured (hereafter referred to as IDD‑NP cells). Another six NP samples were obtained from trauma patients without disc degeneration, and the corresponding NP cells were isolated and cultured as controls (control‑NP cells). The human NP cells were cultured as described in previous studies [23, 24]. Stable overexpression vector (oe‐OIP5, oe‐DEPDC1) and empty vector (used as a negative control) were transfected via LipoFectamine‐2000 (Invitrogen, Carlsbad, CA, USA) into NP cells of IDD upon reaching 90% confluence.

### 2.11. Cell Counting Kit‐8 (CCK‐8) Assay

The viability of the cells was determined using a CCK‐8 kit (Beyotime Biotechnology, Shanghai, China), and the assay was performed according to the manufacturer’s guidelines. NP cells from all groups were seeded into 96‐well plates at a concentration of 5 × 10^3^ cells/well and incubated for 0, 24, 48, and 72 h. At each designated time interval, 10 μL of CCK‐8 reagent was dispensed into the wells, followed by a 1 h incubation period. The absorbance (optical density [OD]) was subsequently quantified at 450 nm.

### 2.12. Terminal Deoxynucleotidyl Transferase‐Mediated dUTP Nick‐End Labeling (TUNEL) Staining

TUNEL staining was performed to detect NP cell apoptosis using the One Step TUNEL Apoptosis Assay Kit (green fluorescence, Beyotime). Specifically, 4% paraformaldehyde was applied to the NP cells for 30 min to achieve fixation. The cells were subsequently treated with 0.3% Triton X‐100 diluted in PBS at room temperature for 5 min to enhance permeability. Thereafter, 50 μL biotin‐labeled solution was applied to each sample and incubated for 60 min at 37°C in the dark. Finally, the samples were mounted with an antifade mounting medium (Beyotime) and visualized under a fluorescence microscope.

### 2.13. Real‐Time Quantitative PCR (RT‐qPCR)

Following the protocol of the Trizol reagent (Thermo Fisher Scientific, Waltham, MA, USA), we isolated total RNA from the cultured cells. Next, the purified RNA underwent reverse transcription via a TaKaRa kit (Tokyo, Japan). Last, gene expression was measured by q‐PCR with the 2 × Cham Q SYBR qPCR Master Mix (Vazyme, Nanjing, Jiangsu, China), employing gene‐specific primers: OIP5: CGCCCTTCCTAGTTGGCATT, CAGGGCAGCATGGGTAGAAT; DEPDC1: AGTGCCTTCTTCTCAGTCTGC, AGCTGCATTTTCTTAGCACATCT; and GAPDH: GACAGTCAGCCGCATCTTCT, GCGCCCAATACGACCAAATC. GAPDH was selected as an internal standard for normalization. The 2^–∆∆Ct^ method was applied to determine relative quantification [25].

### 2.14. Western Blot

Cellular proteins were isolated by applying RIPA lysis buffer (Applygen, Beijing, China), which was mixed with protease and phosphatase inhibitors (Beyotime). Following quantification of protein concentrations via the BCA assay (Beyotime), samples were separated by SDS‐PAGE and electroblotted onto PVDF membranes (Millipore, Merck KGaA, Darmstadt, Germany). After blocking with 5% BSA in TBST (0.1% tris‐buffered saline with Tween 20) at room temperature for 2 h, the membranes were probed overnight with anti‐OIP5 (Proteintech, Wuhan, China), anti‐DEPDC1 (Abcam, Cambridge, UK), and anti‐GAPDH (Proteintech) at 4°C. After three washes with TBST, we incubated the membranes with HRP‐conjugated secondary antibodies (Proteintech) for 1 h at room temperature. Using GAPDH as a loading control, protein bands were detected by ECL (Millipore).

### 2.15. Statistical Analysis

All results are presented as mean ± standard deviation (SD) derived from three independent experiments. Statistical analyses were carried out using SPSS 18.0 and GraphPad Prism 8.0. Both the normality of data distribution and the homogeneity of variances were examined using the Shapiro–Wilk test and Levene’s test, respectively. A two‐tailed Student’s *t*‐test was applied for comparisons between two groups when the assumptions of normality and equal variance were satisfied. If not, the Mann–Whitney *U* test was used. For multiple group comparisons, one‐way ANOVA followed by Bonferroni post hoc correction for multiple testing was employed when parametric assumptions held. If the assumptions were violated, the Kruskal–Wallis test with Dunn’s post hoc correction was performed. Statistical significance was set at *p* < 0.05.

## 3. Results

### 3.1. Separate Identification Yielded 2123 DEGs and 10,695 Key Module Genes

**Figure 1:**
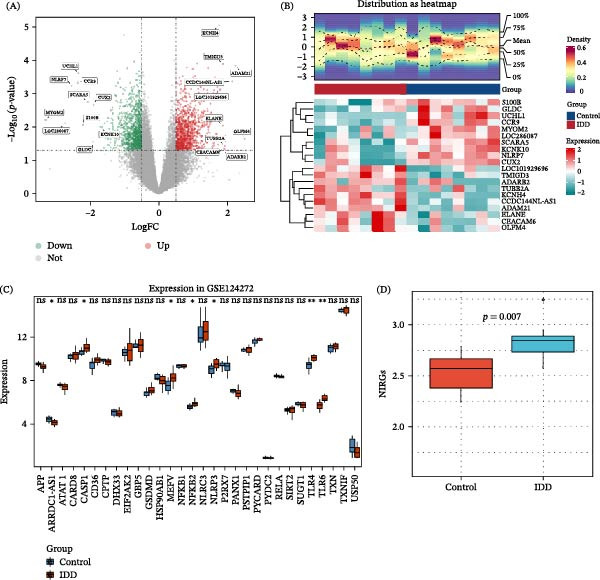
The 2123 DEGs were separately identified. (A) Volcano map of DEGs between control and IDD samples in GSE124272 dataset. (B) Heatmap of DEGs between control and IDD samples in GSE124272 dataset. (C) Box plot of DE‐NIRGs between control and IDD samples through the Wilcoxon test. (D) The difference of NIRGs scores between IDD and control samples in GSE124272 dataset was analyzed using Wilcoxon test.  ^∗^ *p* < 0.05,  ^∗∗^ *p* < 0.01.

**Figure 2:**
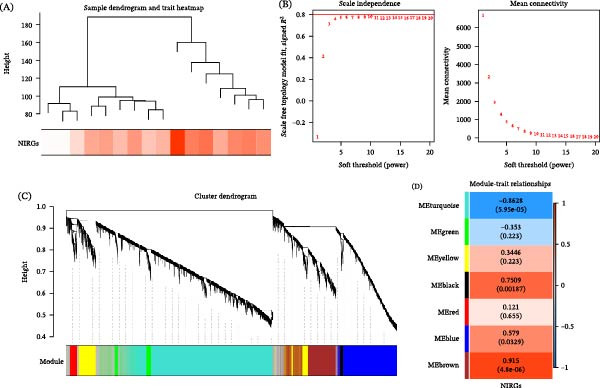
The 10,695 key module genes were separately identified. (A) Sample dendrogram and trait heatmap. (B) Scale independence and mean connectivity. (C) Gene dendrogram and module colors. (D) Module‐trait relationships.

Upregulated genes accounted for 1044 of the 2123 DEGs, while downregulated genes accounted for the remaining 1079 (Figure 1A, B). Furthermore, 30 NIRGs were obtained from the GO and Reactome databases. Seven DE‐NIRGs were obtained by intersecting the DEGs with the NIRGs, namely, ARRDC1‐AS1, CASP1, MEFV, NFKB2, NLRP3, TLR4, and TLR6 (Figure 1C). Later, the NIRGs scores were significantly higher in IDD samples compared with controls (*p* = 0.007), IDD samples had a higher NIRGs scores (Figure 1D). In addition, no outlier samples were identified (Figure 2A). The mean connectivity approached 0, and a soft‑thresholding power of 7 was selected as it was the lowest value at which the scale‑free fit index (*R* ^2^) first reached 0.8 (Figure 2B). Then, a coexpression matrix was established, and seven gene modules were identified (Figure 2C). The MEturquoise and MEbrown modules, which had high correlation with NIRGs score, were selected as key modules (Figure 2D). The 10,695 genes of MEturquoise (8218 genes) and MEbrown (2477 genes) were considered the core module genes.

### 3.2. Chromosomal Distribution of Enrichment Analysis Results for Intersection Genes and 31 Hub Genes

**Figure 3:**
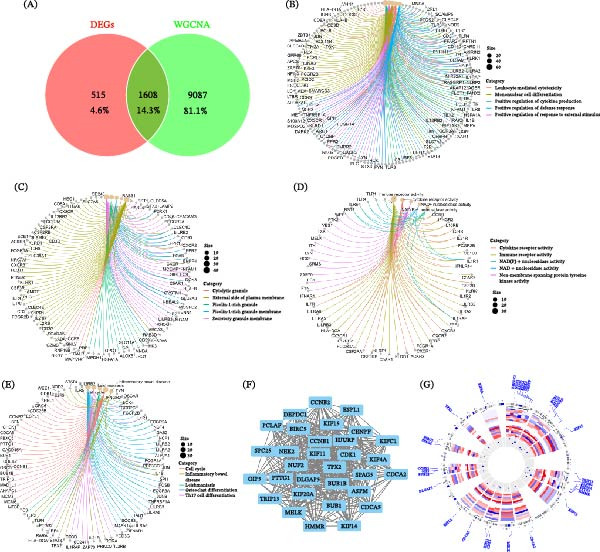
The 1608 intersection genes were mainly enriched in cell cycle pathway and the 31 hub genes were distributed across different chromosomes. (A) Overlapping genes between 2123 DEGs and 10,695 key module genes. (B–D) GO analysis. (E) KEGG analysis. (F) PPI network of 31 hub genes. (G) The locations of hub genes on chromosomes were individually identified using “RCircos” package.

A total of 1608 overlapping genes were identified by intersecting the 2123 DEGs and 10695 core module genes (Figure 3A). According to GO enrichment analysis, these genes showed significant enrichment in terms, including but not limited to leukocyte‐mediated cytotoxicity, cytolytic granules, and cytokine receptor activity (Figure 3B–D). KEGG pathway analysis indicated that they were associated with the cell cycle, inflammatory bowel disease, leishmaniasis, and so on (Figure 3E). Moreover, the interaction network constructed from these genes contained 684 nodes connected by 2327 edges. Then, by MCODE screening, the 31 hub genes from the highest‐scoring cluster were identified (Figure 3F). Examples include HMMR‐KIF14 and BUB1‐ASPM interactions. Additionally, the chromosome locations of the core genes were mapped, excluding PCLAF; they were distributed across chromosomes 1, 2, 3, 5, 6, 8, 9, 10, 11, 12, 14, 15, 17, 20, and X (Figure 3G). Specifically, DEPDC1, NUF2, ASPM, KIF14, NEK2, and CENPF were located on chromosome 1, while BUB1, SPC25, and HJURP were found on chromosome 2, and so forth.

### 3.3. The DEPDC1 and OIP5 Were Deemed as Biomarkers

**Figure 4:**
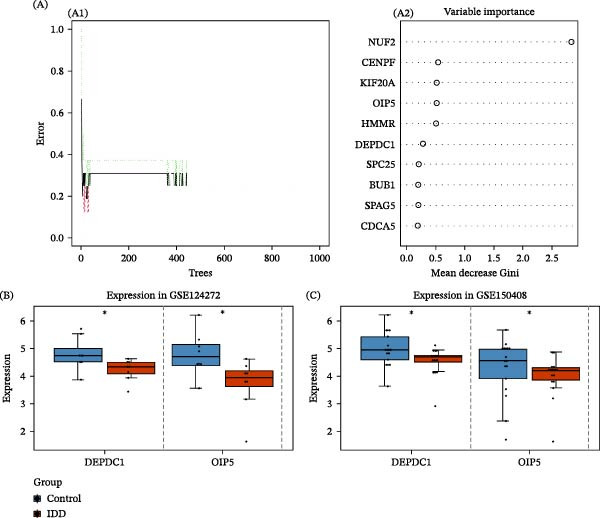
The DEPDC1 and OIP5 were deemed as biomarkers. (A) An out‐of‐bag (OOB) error curve was generated to visualize how the error rate changes as the number of trees increases in the model (A1). Variable importance plot, highlighting the top 10 most important genes ranked by their contribution to mean decrease Gini (A2). (B) Differential expression of DEPDC1 and OIP5 between control and IDD groups in GSE124272 dataset. (C) Differential expression of DEPDC1 and OIP5 between control and IDD groups in GSE150408 dataset.  ^∗^ *p* < 0.05.

Through random forest analysis, the top 10 genes were identified based on their mean decrease Gini, including NUF2, CENPF, KIF20A, OIP5, HMMR, DEPDC1, SPC25, BUB1, SPAG5, and CDCA5 (Figure 4A). Because the expression of DEPDC1 and OIP5 was significantly lower in IDD samples compared to controls in both GSE124272 and GSE150408 datasets (Figure 4B,C), they were designated as biomarkers.

### 3.4. The Nomogram Constructed Based on Two Biomarkers Showed Excellent Performance

Using two biomarkers, a nomogram was generated (Figure S1A). The calibration plot demonstrated good predictive performance, with a HL test result (Figure S1B, *p* = 0.475), and DCA showed a favorable net benefit (Figure S1C).

### 3.5. The Biomarkers Were Mainly Enriched in Cytosolic Ribosome and DNA Replication

GSEA revealed that DEPDC1 and OIP5 are in the cytosolic ribosome, large ribosomal subunit, and ribosomal subunit based on the c5.go.symbols.gmt reference gene set (Figure S2A–B). Additionally, employing the c2.kegg.symbols.gmt as the reference gene set, these two genes were enriched in DNA replication, Parkinson’s disease, proteasome, and so on (Figure S2C–D).

### 3.6. Dysregulation of Immune Cells in IDD and Its Association With Key Biomarkers

**Figure 5:**
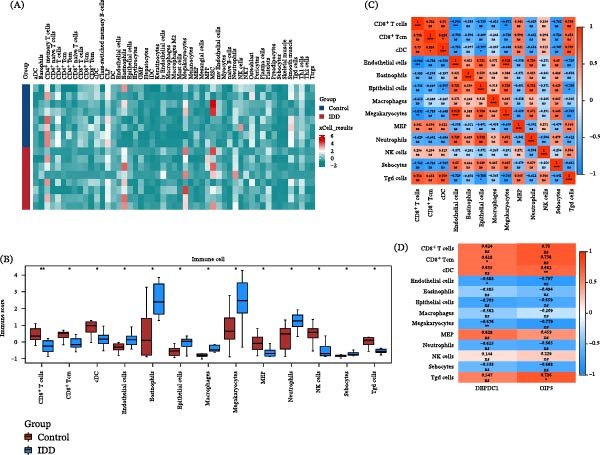
Dysregulation of immune cells in IDD and its association with key biomarkers. (A) Immune cell infiltration in control and IDD groups. (B) Expression differences of various immune cells between the control and IDD groups. (C) Correlation between immune cells and immune cells. (D) Correlation between biomarkers and immune cells.  ^∗^ *p* < 0.05,  ^∗∗^ *p* < 0.01,  ^∗∗∗^ *p* < 0.001.

After excluding immune cells with zero abundance in all samples (Figure 5A), we identified 13 immune cells with significantly different abundance between IDD and control samples (*p* < 0.05), including a lower immune score for CD8^+^ T cells in IDD samples, while eosinophils showed a higher score in IDD samples (Figure 5B). In addition, correlation analysis revealed that CD8^+^ Tcm exhibited the most pronounced positive relationship with conventional dendritic cells (CDC) (*r* = 0.824, *p* < 0.05), while CD8^+^ T cells showed the strongest inverse correlation with (*r* = −0.906, *p* < 0.001) (Figure 5C). Furthermore, analysis of the relationship between biomarkers and differential immune cells showed that DEPDC1 was positively correlated with CD8^+^ Tcm (*r* = 0.618, *p* < 0.05) but negatively related to endothelial cells (*r* = −0.688, *p* < 0.05), whereas OIP5 displayed a marked positive connection with CDC (*r* = 0.662, *p* < 0.01) (Figure 5D).

### 3.7. In Silico Prediction of TP53, CUL1, and CENPA as Potential Upstream Regulators of the Identified Biomarkers

**Figure 6:**
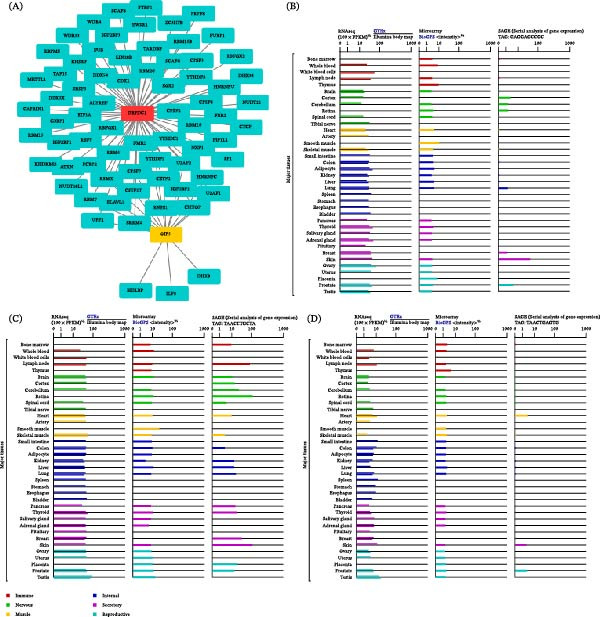
The TP53, CUL1, and CENPA, played a significant role in the expression and regulation of these biomarkers. (A) Biomarkers‐RBP network. (B–D) TP53, CUL1, and CENPA involved in the expression and regulation of biomarkers based on the related information of biomarkers of GeneCards database.

**Figure 7:**
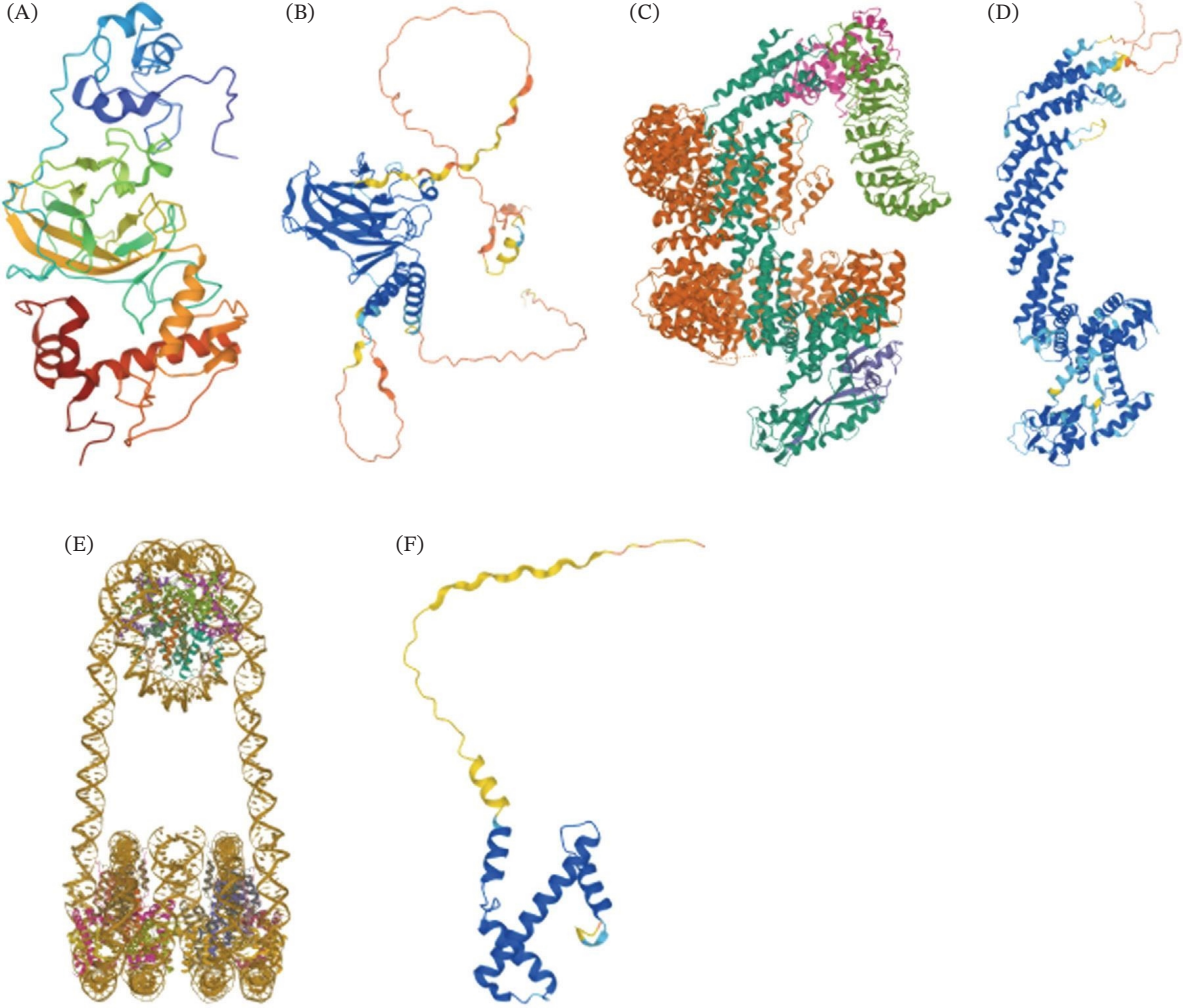
The constructs of biomarkers from the PDB and AlphaFold. (A, B) The constructs of TP53 from the PDB and AlphaFold. (C, D) The constructs of CUL1 from the PDB and AlphaFold. (E, F) The constructs of CENPA from the PDB and AlphaFold.

As an exploratory analysis, we used RBP databases to predict proteins that may bind to the identified biomarkers. The top five proteins were ELAVL1, RBMX, HNRNPC, U2AF2, and TARDB; a biomarkers‐RBP network comprising 73 nodes and 84 edges was subsequently constructed (Figure 6A). Additionally, through querying the GeneCards database, we retrieved three proteins—TP53, CUL1, and CENPA—as candidate upstream regulators based on their high relevance scores to the biomarkers (Figure 6B–D). Structural models of these proteins from the PDB and AlphaFold are shown in Figure 7A–F. These computational predictions are preliminary and require experimental validation to confirm their biological relevance in IDD.

### 3.8. Validation of Decreased OIP5 and DEPDC1 Expression in IDD Clinical Specimens and NP Cells

**Figure 8:**
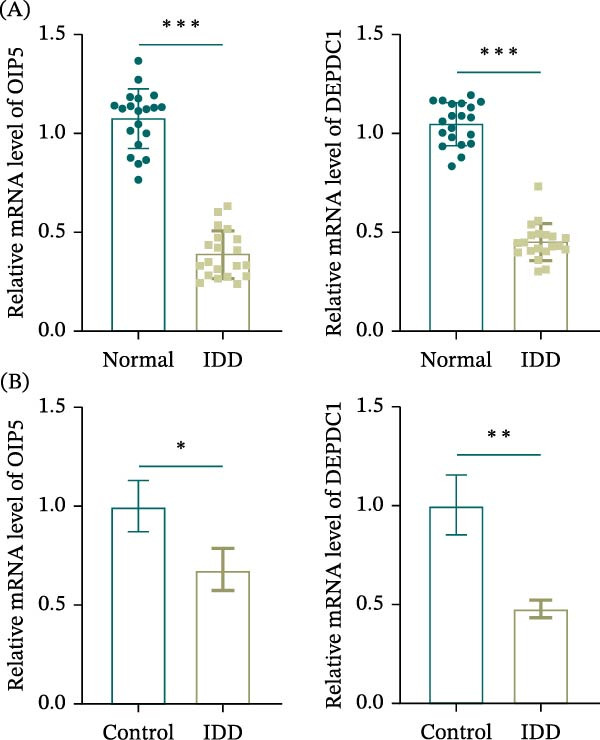
The mRNA expression of two hub genes in tissues and cells of IDD patients. (A) The mRNA expression of OIP5 and DEPDC1 in IDD and normal tissue. (B) The mRNA expression of OIP5 and DEPDC1 in NP cells in IDD and control.  ^∗^ *p* < 0.05,  ^∗∗^ *p* < 0.01,  ^∗∗∗^ *p* < 0.001.

With the aim of validating the expression patterns of the two core genes, OIP5 and DEPDC1, and the disease progression of IDD patients, we examined the expression of these two genes in IDD tissues and an in vitro model, respectively. The results revealed that the mRNA levels of OIP5 and DEPDC1 in IDD tissues were about half the levels of those in normal control tissues (obtained from trauma patients) (*p* < 0.001, Figure 8A). Western blot results showed the same trend (Figure S3A–B). For in vitro model, the expression of OIP5 and DEPDC1 in NP cells of IDD patients was inhibited by about 50% (*p* < 0.05, Figure 8B). Western blot results also showed that the protein expression of OIP5 and DEPDC1 was decreased in the NP cells of IDD patients (Figure S4).

### 3.9. Overexpression of OIP5 Inhibited Proliferation and Promoted Apoptosis of NP Cells in IDD Patients

**Figure 9:**
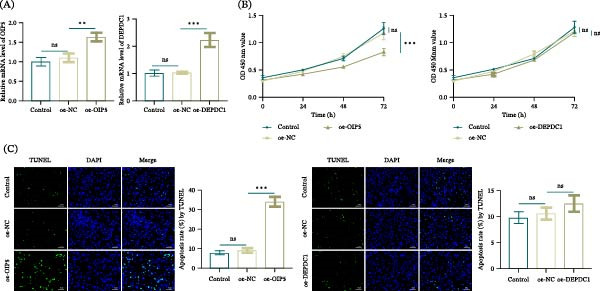
OIP5 inhibited cell proliferation and promoted apoptosis for NP cells in IDD. (A) RT‐qPCR was used to measure the overexpression efficiency of OIP5 and DEPDC1 in NP cells. (B) Cell viability detection of NP cells in different groups by CCK‐8 kit. (C) TUNEL staining was used to measure the apoptosis rate of NP cells. ns, represents no difference,  ^∗∗^ *p* < 0.01,  ^∗∗∗^ *p* < 0.001.

To further evaluate the proliferation ability of OIP5 and DEPDC1 on NP cells in IDD, OIP5‐ and DEPDC1‐overexpressing cell lines were constructed. The transcription levels of OIP5 and DEPDC1 were upregulated by more than about one fold (*p* < 0.01, Figure 9A, Figure S1 C). We subsequently used the CCK‐8 assay to examine whether OIP5 and DEPDC1 overexpression had an effect on cell proliferation. The cell survival rate of the oe‐OIP5 group was reduced by more than half (*p* < 0.001, Figure 9B), whereas the oe‑DEPDC1 group showed no significant difference (*p* > 0.05, Figure 9B), compared with the oe‐NC group. Finally, the apoptotic activity was evaluated by TUNEL staining. Similarly, compared with the normal group, the apoptosis rate in the oe‐OIP5 group increased by ~3‐fold (*p* < 0.001, Figure 9C). The apoptotic activity of DEPDC1 overexpression group had no significant difference compared with the control group (*p* > 0.05, Figure 9C). Taken together, these data demonstrate that OIP5 overexpression could promote the apoptosis of NP cells in IDD.

## 4. Discussion

As a major cause of LBP, IDD involves complex physiological changes and intricate underlying mechanisms. However, study on IDD mechanisms are lacking, especially NLRP3 inflammasome. This studies explored potential molecular mechanisms related to IDD by bioinformatics, and DEPDC1 and OIP5 associated with the NLRP3 inflammasome in IDD were identified as biomarkers. In cell proliferation, migration, and invasion, DEPDC1 is considered a cancer/testis antigen (CTA) and plays significant roles [26]. Among 33 human cancer types, 29 display substantial overexpression of DEPDC1, whose expression levels are additionally associated with clinical outcomes, including survival and prognosis [27]. In integrated bioinformatics analysis focusing on IDD, DEPDC1 clearly separated degenerative IVD tissues from healthy tissue data. Additionally, DEPDC1 was downregulated in degenerative NPCs [28]. Consistent with the above research results, DEPDC1 expression was significantly lower in IDD compared to control samples in GSE124272 and GSE150408 datasets in this study.

OIP5 belongs to the CTA family and is crucial for the maintenance of the centromere structure and function [29]. Dysregulation of OIP5 has been observed in various cancer types; for example, upregulation of OIP5 is markedly correlated with poor prognosis of lung cancer and esophageal cancer, and it is also a prognostic biomarker and a potential target for treatment [30]. However, at present, a detailed understanding of the role of OIP5 in IDD is lacking. In a genetic crossover study of IDD and its associated risk factors, OIP5 was identified as the central protein [31]. Furthermore, OIP5 was identified as the hub gene, which was downregulated in IDD [32]. Consistent with the above research results, OIP5 expression was significantly lower in IDD compared to controls in both GSE124272 and GSE150408 datasets in this study. In addition, compared with the normal group, we discovered through clinical sample testing that the levels of OIP5 were down‐regulation in the IDD group. The above reports and the results of this study suggest that OIP5 plays a critical role in the progression of IDD.

Notably, although OIP5 is downregulated in IDD tissues, its overexpression in NP cells derived from IDD patients inhibits proliferation and promotes apoptosis. As a cell cycle‐related gene, there may be a nonlinear relationship between the expression level and the function of OIP5. OIP5 at the physiological level maintains normal cell proliferation, and long‐term low expression may be implicated in the chronic pathogenesis of IDD through cumulative effects, but acute overexpression to the superphysiological level may activate the apoptotic pathway [33–36]. NP cells derived from IDD patients have been in the pathological microenvironment of inflammatory stress, and their response to gene disturbance may be different from that of normal cells [37–39]. In addition, the time scale difference between chronic low expression and acute overexpression may also lead to different biological effects [40]. Therefore, the exact role of OIP5 in IDD may be characterized by “context dependence,” and its functional differences at different stages and under different intervention methods need to be further studied.

GSEA showed that DEPDC1 and OIP5 were enriched in pathways related to the cytosolic ribosome, large ribosomal subunit, ribosomal subunit, DNA replication, and so on. Cytosolic ribosomes are involved in diverse cellular processes, including protein biosynthesis, cell cycle, DNA repair, cellular proliferation, apoptosis, and autophagy. Through the action of various biogenesis factors, ribosome biogenesis encompasses RNA processing, modification, and binding to ribosomal proteins [41]. DNA replication ensures accurate duplication and transfer of genetic information [42]. Telomeres are composed of repetitive DNA repeat sequences of 5′‐TTAGGG‐3′, which shorten with each replication cycle [43, 44]. Consistent with our bioinformatics predictions, a previous study demonstrated that the number of NP chondrocytes rose with aging and progressive disc degeneration, while telomere shortening and reduced telomerase activity were observed. Oxidative stress involved in many disease processes is also involved in IDD [45, 46]. It is well established that oxidative stress can hamper NP cell proliferation, provoke premature senescence, and promote catabolic phenotype, which is consistent with the replication‐telomere stress‐related clues we have seen. Autophagy is also closely related to these processes. Activation of autophagy has been shown to promote tissue regeneration under mechanical stretch by reducing apoptosis and supporting proliferation [47–49], while caragliflozin reduces disease progression in ApoE−/−atherosclerosis models by coordinating the inhibition of inflammation/oxidative stress and enhancement of autophagy [50]. It has been reported that mitophagy alleviates IDD by inhibiting cGAS‐STING and NLRP3 inflammasome‐mediated pyroptosis pathways [51], which together support the “stress‐autophagy‐cell fate” regulatory framework highly associated with IDD. At the same time, new work on RNA processing has shown that altered intron polyadenylation (for example, reducing the use of GLS intron polyadenylation) can promote cell senescence [52], providing an additional mechanism analogy that posttranscriptional regulation may converge to the senescent phenotype. This further validates the reliability of our research. However, these functional effects are inferred from bioinformatics predictions and require an in‐depth study of direct cell experiments and animal experiments to confirm their correlation in IDD.

Immune infiltration analysis revealed 13 differentially abundant immune cells between IDD and control samples, including decreased CD8^+^ T cells and increased eosinophils in IDD tissues. The IVD is recognized as an immune‐privileged tissue. When the blood–NP barrier is damaged, immune cells enter, and inflammation is amplified. However, different cohorts and disease stages may show dynamic changes and “apparent reduction” of T cell subsets (such as migration to focal tissue or functional exhaustion). This has been suggested in previous studies and reviews of immune infiltration based on transcriptome deconvolution [53, 54]. Meanwhile, we observed a strong association between CD8^+^ Tcm and CDC, which also conforms to the immunological law of CDC maintaining/shaping CD8 memory T cell homeostasis and response, suggesting that there may be a synergistic change of antigen presentation‐memory T cell axis in degenerative tissues [55]. Eosinophils are elevated in IDD. Studies have reported that eosinophils can inhibit osteoclast differentiation and bone resorption and have a protective effect in inflammatory bone loss models [56]. However, its functional significance in IDD remains to be fully elucidated. In addition, we detected a marked inverse relationship between CD8^+^ T cells and endothelial cells. Combined with the view that vascular endogenesis of degenerative IVD promotes the entry of immune cells, it is suggested that “vascular‐immune” coupling may be involved in the rearrangement and focal aggregation of immune cell lineage [57]. Moreover, DEPDC1 had an obviously positive correlation with CD8^+^ Tcm while it had a negative correlation with endothelial cells. OIP5 and CDC had significantly positive relationship. According to the report, down‐regulation of DEPDC1 suppressed the tube formation of endothelial cells [58], this is consistent with the direction of our correlation results. Of course, these inferences are all based on the prediction results of this study and other people’s research, and we need to further study in the future based on cell experiments and animal experiments.

In the present study, NP cells from IDD patients in IDD were used to establish OIP5 and DEPDC1 overexpression cells, respectively. We found that overexpression inhibited cell proliferation, whereas DEPDC1 overexpression had no significant effect on NP cell proliferation. These findings suggest that OIP5 may play an inhibitory role in the progression of IDD, although the underlying mechanisms—including whether this effect is mediated through the pathways predicted by GSEA—require direct experimental investigation.

Nevertheless, certain limitations of this work should be noted. First, the discovery cohorts (GSE124272 and GSE150408) are based on whole blood transcriptome data, which primarily reflect systemic changes associated with IDD rather than the molecular characteristics of the local disc tissue; although subsequent experimental validation using disc tissue samples confirmed the expression of OIP5, the limited sample size may hinder our ability to generalize these findings. Second, the underlying mechanisms between NLRP3 inflammasome and immune infiltration deserve further research. Third, functional experiments were performed only in IDD‐derived NP cells, which limited a comprehensive understanding of the role of OIP5 in the pathogenesis of IDD and could not support any claims about immune regulation due to the lack of a corresponding immune microenvironment in cell experiments. Subsequently, OIP5 overexpression/knockdown control in normal NP cells and stratified verification under inflammatory stimulation (such as IL‐1β/TNF‐α) and nonstimulation conditions were required; further exploration in 3D/explants or animal models was closer to the tissue microenvironment. Fourth, although the nomogram shows acceptable calibration and clinical net benefit in the pooled data set, these findings are exploratory and need to be further verified. Future studies should use independent cohorts to evaluate the universality of the prediction model. In addition, cross‐validation, or split‐sample validation in a larger dataset will provide a more robust assessment of model performance. Fifth, although RT‐qPCR was applied to assess the mRNA expression of OIP5 and DEPDC1 in clinical specimens and in vitro models, due to limited sample availability, protein levels were not confirmed by western blot or immunohistochemistry. In view of the active posttranscriptional regulation in degenerative tissues, future studies should be combined with protein‐level analysis to confirm these findings. Finally, many results in this study are based on bioinformatics database mining, such as TP53, CUL1, and CENPA as upstream regulators of OIP5 and DEPDC1. These results need to be further verified in cell and animal experiments.

## 5. Conclusion

In conclusion, using whole blood transcriptome data, the present study found two systemic biomarkers (DEPDC1 and OIP5) for IDD through differential expression analysis, WGCNA, PPI network, and machine learning. In addition, GSEA enrichment analysis of biomarkers was conducted and found that they were mainly involved in pathways, e.g., cytoplasmic ribosomes and DNA replication. Immune infiltration analysis and drug prediction analyses were performed, and 13 differentially expressed immune cells were identified. Finally, this study confirmed that OIP5 was expressed at a low level in the tissues of patients with IDD and inhibited the proliferation of NP cells in IDD patients. These findings provide a systemic perspective on IDD‐related inflammation and identify potential therapeutic targets warranting further investigation.

**Nomenclature**
IDD: Intervertebral disc degeneration
DEGs: Differentially expressed genes
ssGSEA: Single‐sample gene set enrichment analysis
WGCNA: Weighted gene coexpression network analysis
PPI: Protein–protein interaction
LBP: Low back pain
IVD: Intervertebral disc
NP: Nucleus pulposus
ECM: Extracellular matrix
NLRP3: Nucleotide‐binding oligomerization domain‐like receptor pyrin domain‐containing 3
ASC: Adapter apoptosis‐associated speck‐like protein containing a C‐terminal caspase recruitment domain
NIRGs: NLRP3 inflammasome‐related genes
GO: Gene Ontology
FC: Fold change
KEGG: Kyoto Encyclopedia of Genes and Genomes
DCA: Decision curve analysis
HL: Hosmer–Lemeshow
MSigDB: Molecular signatures database
RBP: RNA binding protein
PDB: Protein Data Bank
CTD: Comparative toxicogenomics database
CDC: Conventional dendritic cells
DEPDC1: Disheveled, EGL‐10, and pleckstrin domain‐containing 1
CTA: Cancer/testis antigen.

## Author Contributions

All authors significantly contributed to various aspects of the reported work, including conception, study design, execution, data acquisition, analysis and interpretation, drafting, revising, and critical review of the article.

## Funding

This study was supported by Shanxi Graduate Education Innovation Plan Project (Grant 2024L132), the Key Research and Development Program of Linfen City (Grant 2405), the National Natural Science Foundation of China (Grant 82202753), the Fundamental Research Program of Linfen People’s Hospital (Grant nos: T2023097 and T2025005).

## Disclosure

All authors provided final approval for the submitted version and the selected journal, demonstrating their commitment to the complete research process and assuming accountability.

## Ethics Statement

Studies involving humans were approved by the ethics committee of Linfen People’s Hospital (Number E20231128083), and complied with the Declaration of Helsinki. All participants provided written informed consent to participate in this study.

## Conflicts of Interest

The authors declare no conflicts of interest.

## Supporting Information

Additional supporting information can be found online in the Supporting Information section.

## Supporting information


**Supporting Information** Figure S1: The nomogram constructed based on two biomarkers showed excellent performance. Figure S2: The biomarkers were mainly enriched in cytosolic ribosome and DNA replication. Figure S3: The protein expression of two hub genes in tissues and cells of IDD patients. Figure S4: Western blot was used to measure the overexpression efficiency of OIP5 and DEPDC1 in NP cells.

## Data Availability

All the results are presented in the article. Further inquiries can be directed to the corresponding authors.

## References

[1] GBD , Global Burden of 369 Diseases and Injuries in 204 Countries and Territories, 1990–2019: A Systematic Analysis for the Global Burden of Disease Study 2019, 2020, 396, no. 10258, 1204–1222, 10.1016/S0140-6736(20)30925-9. PMC7567026 33069326

[2] Vergroesen P. P. , Kingma I. , and Emanuel K. S. , et al.Mechanics and Biology in Intervertebral Disc Degeneration: A Vicious Circle, Osteoarthritis and Cartilage. (2015) 23, no. 7, 1057–1070, 10.1016/j.joca.2015.03.028. 25827971

[3] Li G. , Zhang W. , Liang H. , and Yang C. , Epigenetic Regulation in Intervertebral Disc Degeneration, Trends in Molecular Medicine. (2022) 28, no. 10, 803–805, 10.1016/j.molmed.2022.07.007. 36030154

[4] Song C. , Zhou Y. , and Cheng K. , et al.Cellular Senescence - Molecular Mechanisms of Intervertebral Disc Degeneration From an Immune Perspective, Biomedicine & Pharmacotherapy. (2023) 162, 10.1016/j.biopha.2023.114711, 114711. 37084562

[5] Wang Z. , Hu X. , and Cui P. , et al.Progress in Understanding the Role of cGAS-STING Pathway Associated With Programmed Cell Death in Intervertebral Disc Degeneration, Cell Death Discovery. (2023) 9, no. 1, 10.1038/s41420-023-01607-7, 377. 37845198 PMC10579269

[6] Zehra U. , Tryfonidou M. , Iatridis J. C. , Illien-Jünger S. , Mwale F. , and Samartzis D. , Mechanisms and Clinical Implications of Intervertebral Disc Calcification, Nature Reviews Rheumatology. (2022) 18, no. 6, 352–362, 10.1038/s41584-022-00783-7. 35534553 PMC9210932

[7] Hooftman A. , Angiari S. , and Hester S. , et al.The Immunomodulatory Metabolite Itaconate Modifies NLRP3 and Inhibits Inflammasome Activation, Cell Metabolism. (2020) 32, no. 3, 468–478, 10.1016/j.cmet.2020.07.016. 32791101 PMC7422798

[8] Zhao C. , Zheng T. , and Wang R. , et al.Synergistically Augmenting Cancer Immunotherapy by Physical Manipulation of Pyroptosis Induction, Phenomics. (2024) 4, no. 3, 298–312, 10.1007/s43657-023-00140-y. 39398428 PMC11466912

[9] Fu J. and Wu H. , Structural Mechanisms of NLRP3 Inflammasome Assembly and Activation, Annual Review of Immunology. (2023) 41, no. 1, 301–316, 10.1146/annurev-immunol-081022-021207. PMC10159982 36750315

[10] Lv H. , Wang Y. , and Tan L. , et al.Erratum to “Interleukin-4-Mediated NLRP3 Inflammasome Activation in Microglia Contributes to Allergic Rhinitis via Central Sensitization, Research. (2026) 9, 10.34133/research.1066, 1066. 41541566 PMC12799906

[11] Tamboli A. R. , Yadav V. D. , and Nadaf S. J. , et al.Exploring the Frontier of Inhalation Therapy: A Review of Dry Powder Inhalers for Precision Management of Inflammatory Lung Diseases, BIO Integration. (2024) 5, no. 1, 10.15212/bioi-2024-0062, 962.

[12] Sharma B. R. and Kanneganti T. D. , NLRP3 Inflammasome in Cancer and Metabolic Diseases, Nature Immunology. (2021) 22, no. 5, 550–559, 10.1038/s41590-021-00886-5. 33707781 PMC8132572

[13] Chao-Yang G. , Peng C. , and Hai-Hong Z. , Roles of NLRP3 Inflammasome in Intervertebral Disc Degeneration, Osteoarthritis and Cartilage. (2021) 29, no. 6, 793–801, 10.1016/j.joca.2021.02.204. 33609693

[14] Song Y. , Wang Y. , and Zhang Y. , et al.Advanced Glycation End Products Regulate Anabolic and Catabolic Activities via NLRP3-Inflammasome Activation in Human Nucleus Pulposus Cells, Journal of Cellular and Molecular Medicine. (2017) 21, no. 7, 1373–1387, 10.1111/jcmm.13067. 28224704 PMC5487914

[15] Gustavsson E. K. , Zhang D. , Reynolds R. H. , Garcia-Ruiz S. , and Ryten M. , Ggtranscript: An R Package for the Visualization and Interpretation of Transcript Isoforms Using ggplot2, Bioinformatics. (2022) 38, no. 15, 3844–3846, 10.1101/2022.03.28.486050. 35751589 PMC9344834

[16] Tarnowski M. , Tomasiak P. , Tkacz M. , Zgutka K. , and Piotrowska K. , Epigenetic Alterations in Sports-Related Injuries, 2022, 13, no. 8, 10.3390/genes13081471, 1471. PMC9408207 36011382

[17] Hänzelmann S. , Castelo R. , and Guinney J. , GSVA: Gene set Variation Analysis for Microarray and RNA-Seq Data, BMC Bioinformatics. (2013) 14, no. 1, 10.1186/1471-2105-14-7, 7. 23323831 PMC3618321

[18] Dang Y. , He Q. , and Yang S. , et al.FTH1- and SAT1-Induced Astrocytic Ferroptosis Is Involved in Alzheimer’s Disease: Evidence From Single-Cell Transcriptomic Analysis, Pharmaceuticals. (2022) 15, no. 10, 10.3390/ph15101177, 1177. 36297287 PMC9610574

[19] Doncheva N. T. , Morris J. H. , Gorodkin J. , and Jensen L. J. , Cytoscape StringApp: Network Analysis and Visualization of Proteomics Data, Journal of Proteome Research. (2019) 18, no. 2, 623–632, 10.1021/acs.jproteome.8b00702. 30450911 PMC6800166

[20] Liu T. T. , Li R. , and Huo C. , et al.Identification of CDK2-Related Immune Forecast Model and ceRNA in Lung Adenocarcinoma, a Pan-Cancer Analysis, Frontiers in Cell and Developmental Biology. (2021) 9, 10.3389/fcell.2021.682002, 682002. 34409029 PMC8366777

[21] Liu F. , Shi D.-M. , Ma W.-Y. , Tang D.-W. , Bai G. , and Yu X.-Y. , Targeting CXCR4 and EDN1 for the Treatment of Recurrent Miscarriage Using Stearic Acid From Traditional Chinese Medicine, Traditional Medicine Research. (2024) 9, no. 11, 66–67, 10.53388/TMR20240621002.

[22] Du Y. and Liu H. , Exercise-Induced Modulation of miR-149-5p and MMP9 in LPS-Triggered Diabetic Myoblast ER Stress: Licorice Glycoside E as a Potential Therapeutic Target, Traditional Medicine Research. (2024) 9, no. 8, 45–48, 10.53388/TMR20230121002.

[23] Guo W. , Mu K. , and Zhang B. , et al.The Circular RNA FAM169A Functions as a Competitive Endogenous RNA and Regulates Intervertebral Disc Degeneration by Targeting miR-583 and BTRC, Cell Death & Disease. (2020) 11, no. 5, 10.1038/s41419-020-2543-8, 315. 32366862 PMC7198574

[24] Chen Y. , Wu Y. , Chen R. , Xu C. , and Chen Q. , LncRNA LINC00324 Is Upregulated in Intervertebral Disk Degeneration and Upregulates FasL in Nucleus Pulposus Cells, Molecular and Cellular Biochemistry. (2021) 476, no. 5, 1995–2000, 10.1007/s11010-021-04058-9. 33511550

[25] Li Y. , Wang Z. , and Li H.-N. , et al.Decoding Exercise-Induced Atomic Components and Prognostic Shifts in Endometrial Carcinoma Through Differentially Expressed Genes, Traditional Medicine Research. (2024) 9, no. 11, 61–67, 10.53388/TMR20240402001.

[26] Wang W. , Li A. , and Han X. , et al.DEPDC1 Up-Regulates RAS Expression to Inhibit Autophagy in Lung Adenocarcinoma Cells, Journal of Cellular and Molecular Medicine. (2020) 24, no. 22, 13303–13313, 10.1111/jcmm.15947. 33021072 PMC7701574

[27] Jia B. , Liu J. , Hu X. , Xia L. , and Han Y. , Pan-Cancer Analysis of DEPDC1 as a Candidate Prognostic Biomarker and Associated With Immune Infiltration, Annals of Translational Medicine. (2022) 10, no. 24, 10.21037/atm-22-5598, 1355. 36660720 PMC9843344

[28] Long H. , Liu Q. , and Yin H. , et al.Prevalence Trends of Site-Specific Osteoarthritis From 1990 to 2019: Findings From the Global Burden of Disease Study 2019, Arthritis & Rheumatology. (2022) 74, no. 7, 1172–1183, 10.1002/art.42089. 35233975 PMC9543105

[29] Afsharpad M. , Nowroozi M. R. , and Mobasheri M. B. , et al.Cancer-Testis Antigens as New Candidate Diagnostic Biomarkers for Transitional Cell Carcinoma of Bladder, Pathology & Oncology Research. (2019) 25, no. 1, 191–199, 10.1007/s12253-017-0313-4. 29058301

[30] Koinuma J. , Akiyama H. , and Fujita M. , et al.Characterization of an Opa Interacting Protein 5 Involved in Lung and Esophageal Carcinogenesis, Cancer Science. (2012) 103, no. 3, 577–586, 10.1111/j.1349-7006.2011.02167.x. 22129094 PMC7713625

[31] Xiang H. , Yan F. , and Liu H. , The Genetic Association Identified Between Intervertebral Disc Degeneration and Associated Risk Factors Based on a Systems Biology Approach, Spine. (2022) 47, no. 8, E370–E384, 10.1097/BRS.0000000000004312. 34919076

[32] Fang X. , Tang T. , Sun D. , Chen S. , Wang N. , and Xie L. , Comprehensive Analysis of Potential ceRNA Network and Immune Cell Infiltration in Intervertebral Disc Degeneration, Journal of Orthopaedic Surgery and Research. (2022) 17, no. 1, 10.1186/s13018-022-03331-x, 432. 36175893 PMC9524080

[33] Nardi I. K. , Zasadzińska E. , Stellfox M. E. , Knippler C. M. , and Foltz D. R. , Licensing of Centromeric Chromatin Assembly Through the Mis18α-Mis18β Heterotetramer, Molecular Cell. (2016) 61, no. 5, 774–787, 10.1016/j.molcel.2016.02.014. 26942680 PMC4935545

[34] Pan M. , Wang Y. , and Wang Z. , et al.The Mitosis-Related Gene OIP5 Is a Potential Biomarker in Pan-Cancer, Annals of Translational Medicine. (2023) 11, no. 2, 10.21037/atm-22-6640, 117. 36819584 PMC9929809

[35] Sethi S. C. , Shrestha R. L. , and Balachandra V. , et al.β-TrCP-Mediated Proteolysis of Mis18β Prevents Mislocalization of CENP-A and Chromosomal Instability, Molecular and Cellular Biology. (2024) 44, no. 10, 429–442, 10.1080/10985549.2024.2382445. 39135477 PMC11486186

[36] Zhang X. , Gu W. , and Lin A. , et al.The Role of OIP5 in the Carcinogenesis and Progression of Ovarian Cancer, Journal of Ovarian Research. (2023) 16, no. 1, 10.1186/s13048-023-01265-4, 185. 37660035 PMC10474646

[37] Wang Y. , Che M. , Xin J. , Zheng Z. , Li J. , and Zhang S. , The Role of IL-1β and TNF-α in Intervertebral Disc Degeneration, Biomedicine & Pharmacotherapy. (2020) 131, 10.1016/j.biopha.2020.110660, 110660. 32853910

[38] Zhang Q.-C. , Zou Y.-P. , and Hu S.-Q. , et al.TNF-α-Stimulated Nucleus Pulposus Cells Induce Cell Apoptosis Through the Release of Exosomal miR-16 Targeting IGF-1 and IGF-1R in Rats, Annals of Translational Medicine. (2021) 9, no. 17, 10.21037/atm-21-227, 1376. 34733928 PMC8506555

[39] Wang Z. , Zhu D. , and Yang F. , et al.POSTN Knockdown Suppresses IL-1β-Induced Inflammation and Apoptosis of Nucleus Pulposus Cells via Inhibiting the NF-κB Pathway and Alleviates Intervertebral Disc Degeneration, Journal of Cell Communication and Signaling. (2024) 18, no. 2, 10.1002/ccs3.12030. PMC11208126 38946726

[40] Greenwood S. N. , Belz R. G. , and Weiser B. P. , A Conserved Mechanism for Hormesis in Molecular Systems, Dose-Response. (2022) 20, no. 3, 10.1177/15593258221109335, 15593258221109335. 35936511 PMC9350523

[41] Pecoraro A. , Pagano M. , Russo G. , and Russo A. , Ribosome Biogenesis and Cancer: Overview on Ribosomal Proteins, International Journal of Molecular Sciences. (2021) 22, no. 11, 10.3390/ijms22115496, 5496. 34071057 PMC8197113

[42] Zhu H. , Swami U. , Preet R. , and Zhang J. , Harnessing DNA Replication Stress for Novel Cancer Therapy, Genes. (2020) 11, no. 9, 10.3390/genes11090990, 990. 32854236 PMC7564951

[43] Ghilain C. , Gilson E. , and Giraud-Panis M. J. , Multifunctionality of the Telomere-Capping Shelterin Complex Explained by Variations in Its Protein Composition, Cells. (2021) 10, no. 7, 10.3390/cells10071753, 1753. 34359923 PMC8305809

[44] Gao X. , Yu X. , and Zhang C. , et al.Telomeres and Mitochondrial Metabolism: Implications for Cellular Senescence and Age-Related Diseases, Stem Cell Reviews and Reports. (2022) 18, no. 7, 2315–2327, 10.1007/s12015-022-10370-8. 35460064 PMC9033418

[45] Ding J. , Mei S. , and Wang K. , et al.Curcumin Modulates Oxidative Stress to Inhibit Pyroptosis and Improve the Inflammatory Microenvironment to Treat Endometriosis, Genes & Diseases. (2024) 11, no. 3, 10.1016/j.gendis.2023.06.022, 101053. 38292195 PMC10827401

[46] Hong Y. , Zhou X. , and Li Q. , et al.X-Box Binding Protein 1 Caused an Imbalance in Pyroptosis and Mitophagy in Immature Rats With Di-(2-Ethylhexyl) Phthalate-Induced Testis Toxicity, Genes & Diseases. (2024) 11, no. 2, 935–951, 10.1016/j.gendis.2023.02.030. 37692514 PMC10491871

[47] Du J. , Liu W. , and Song Y. , et al.Activating Autophagy Promotes Skin Regeneration Induced by Mechanical Stretch During Tissue Expansion, Burns & Trauma. (2024) 12, 10.1093/burnst/tkad057, tkad057. 38328438 PMC10849167

[48] Yao J.-Y. , Yang Y.-L. , Chen W.-J. , and Fan H.-Y. , Exploring the Therapeutic Potential of Qi Teng Mai Ning Recipe in Ischemic Stroke and Vascular Cognitive Impairment, Traditional Medicine Research. (2024) 9, no. 10, 57–60, 10.53388/TMR20240214001.

[49] Ding H. , Zeng H. , and Wen Y. , et al.Activation of the α7 Nicotinic Acetylcholine Receptor Mitigates Cognitive Deficits in Mice With Sepsis-Associated Encephalopathy by Inhibiting Microglial Pyroptosis, World Journal of Emergency Medicine. (2025) 16, no. 5, 438–446, 10.5847/wjem.j.1920-8642.2025.099. 40979792 PMC12444236

[50] Zuo Q. , He L. , and Ma S. , et al.Canagliflozin Alleviates Atherosclerosis Progression Through Inflammation, Oxidative Stress, and Autophagy in Western Diet-Fed ApoE−/− Mice, Cardiovascular Innovations and Applications. (2024) 9, no. 1, 10.15212/CVIA.2023.0093, 981.

[51] Wang K. , Wu X. , and Li H. , et al.Mitophagy Reprograms Lactate Metabolism to Suppress THBS1 via H3K18la Reduction, Alleviating Intervertebral Disc Degeneration, Research. (2025) 8, 10.34133/research.0957, 0957. 41199783 PMC12586853

[52] Li X. , Li Y. , and Ding D. , et al.Reduced Usage of GLS Intronic Polyadenylation Promotes Cellular Senescence, Phenomics. (2025) 5, no. 5, 487–501, 10.1007/s43657-024-00198-2. 41659737 PMC12881237

[53] Sun Z. , Liu B. , and Luo Z. J. , The Immune Privilege of the Intervertebral Disc: Implications for Intervertebral Disc Degeneration Treatment, International Journal of Medical Sciences. (2020) 17, no. 5, 685–692, 10.7150/ijms.42238. 32210719 PMC7085207

[54] Wang L. , He T. , and Liu J. , et al.Revealing the Immune Infiltration Landscape and Identifying Diagnostic Biomarkers for Lumbar Disc Herniation, Frontiers in Immunology. (2021) 12, 10.3389/fimmu.2021.666355, 666355. 34122424 PMC8190407

[55] Stonier S. W. , Ma L. J. , Castillo E. F. , and Schluns K. S. , Dendritic Cells Drive Memory CD8 T-Cell Homeostasis via IL-15 Transpresentation, Blood. (2008) 112, no. 12, 4546–4554, 10.1182/blood-2008-05-156307. 18812469 PMC2597127

[56] Andreev D. , Kachler K. , and Liu M. , et al.Eosinophils Preserve Bone Homeostasis by Inhibiting Excessive Osteoclast Formation and Activity via Eosinophil Peroxidase, Nature Communications. (2024) 15, no. 1, 10.1038/s41467-024-45261-8, 1067. PMC10844633 38316791

[57] Sun Z. , Zhao H. , and Liu B. , et al.AF Cell Derived Exosomes Regulate Endothelial Cell Migration and Inflammation: Implications for Vascularization in Intervertebral Disc Degeneration, Life Sciences. (2021) 265, 10.1016/j.lfs.2020.118778, 118778. 33217442

[58] Yang M. , Zhang H. , Gao S. , and Huang W. , DEPDC1 and KIF4A Synergistically Inhibit the Malignant Biological Behavior of Osteosarcoma Cells Through Hippo Signaling Pathway, Journal of Orthopaedic Surgery and Research. (2023) 18, no. 1, 10.1186/s13018-023-03572-4, 145. 36849972 PMC9972622

