# Supplementary material for: Revealing the Correlation Between NLRP3 Inflammasome‐Related Genes and Intervertebral Disc Degeneration Based Multiomic Analysis and Experimental Validation
Source: Mediators Inflamm. 2026 Jul 14;2026:1534745. doi: 10.1155/mi/1534745 (PMC13366407; doi:10.1155/mi/1534745)
Supplement: Supplementary file 1 — Supporting Information Figure S1: The nomogram constructed based on two biomarkers showed excellent performance. Figure S2: The biomarkers were mainly enriched in cytosolic ribosome and DNA replication. Figure S3: The protein expression of two hub genes in tissues and cells of IDD patients. Figure S4: Western blot was used to measure the overexpression efficiency of OIP5 and DEPDC1 in NP cells. [file MI-2026-1534745-s001.docx]

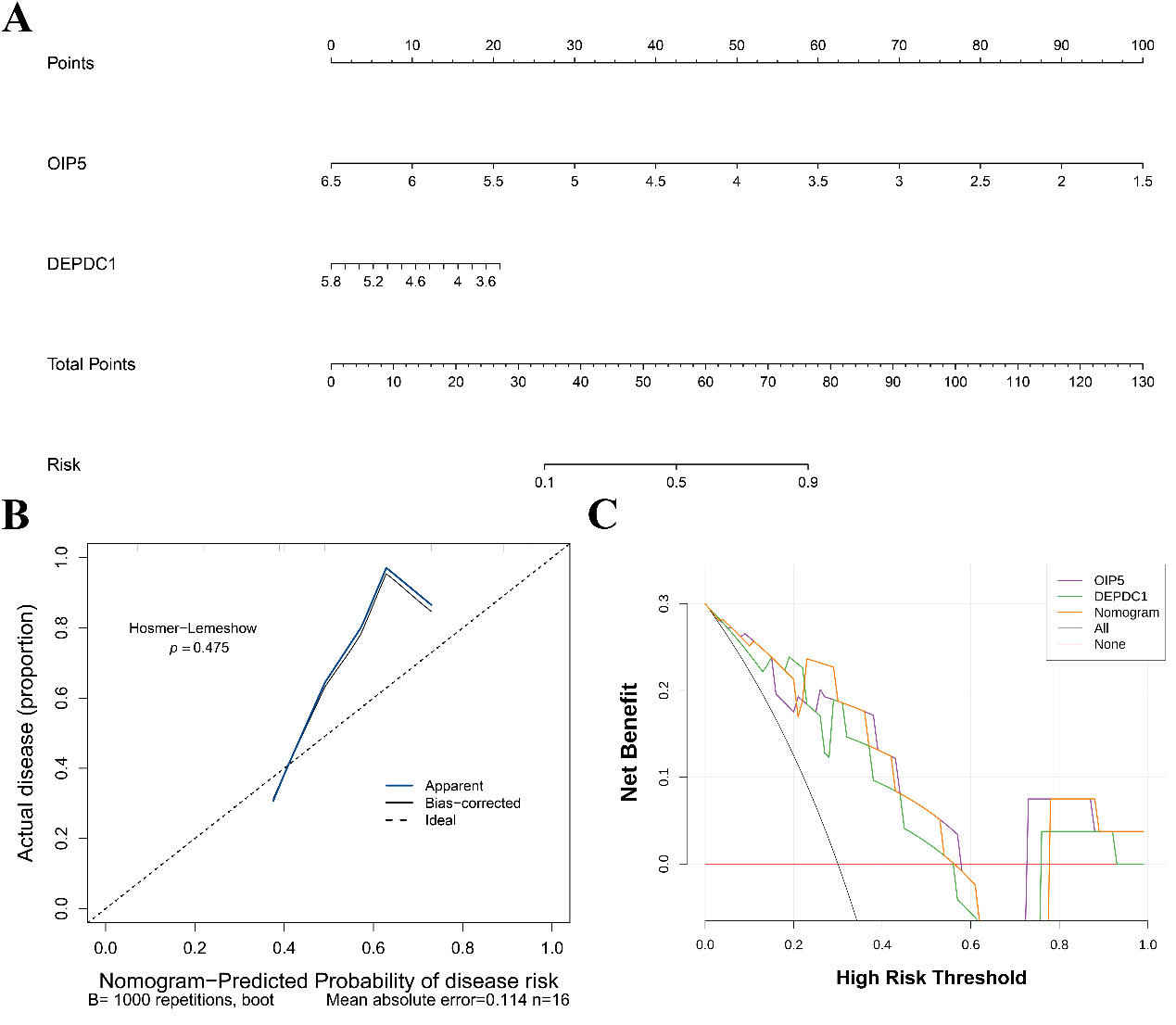
**Figure S1** The nomogram constructed based on 2 biomarkers showed excellent performance. **(A)** The nomogram model was established on the basis of the 2 biomarkers. **(B)** The calibration curve was utilized to evaluate the predictive accuracy of the nomogram model. **(C)** The DCA curve to evaluate the clinical efficiency of the nomogram model.


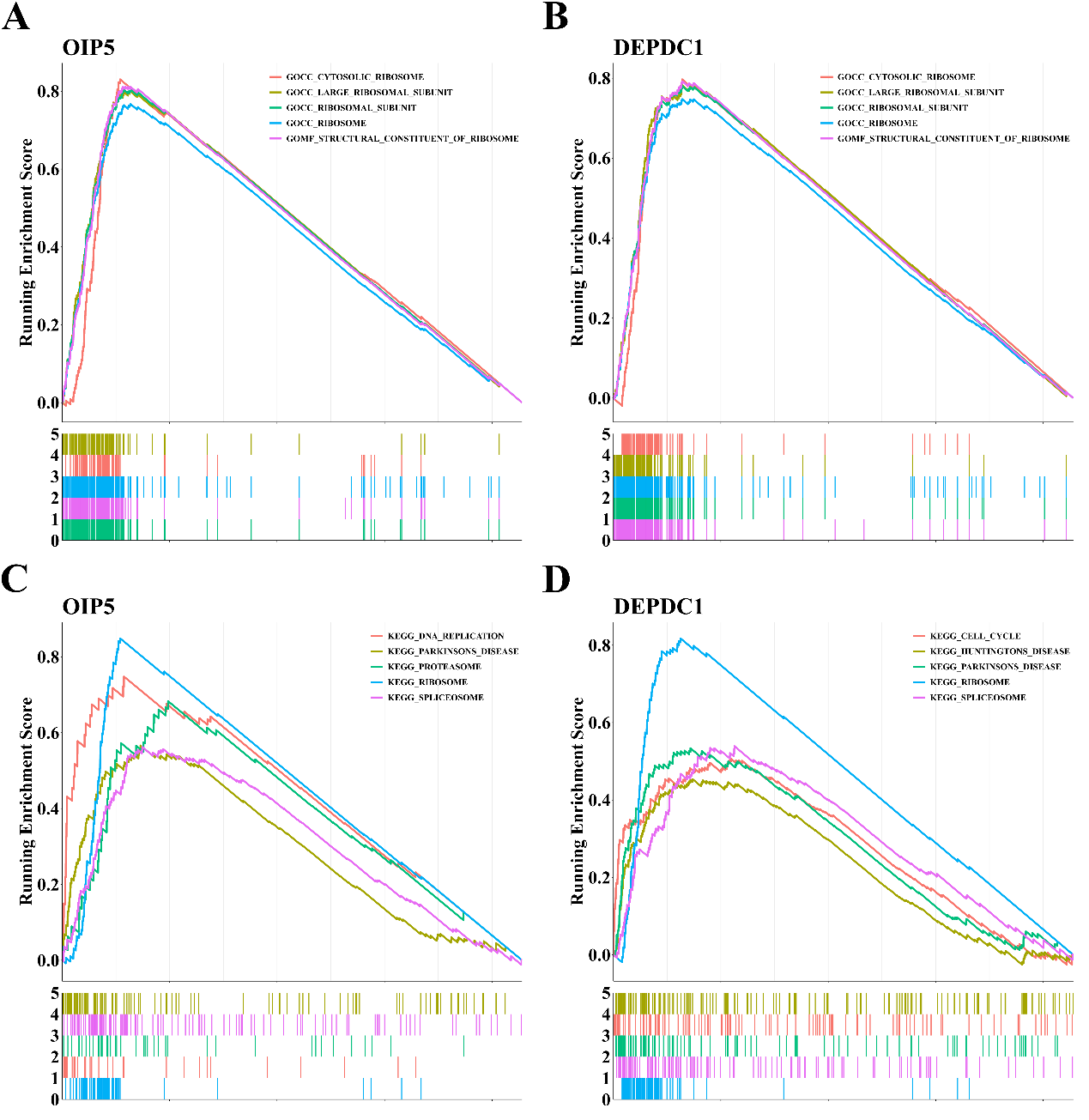


**Figure S2** The biomarkers were mainly enriched in cytosolic ribosome and DNA replication. GSEA of OIP5: **(A)** Analysis of Gene Ontology Cellular Component. **(C)** Analysis of KEGG. GSEA of DEPDC1: **(B)** Analysis of Gene Ontology Cellular Component. **(D)** Analysis of KEGG.

**
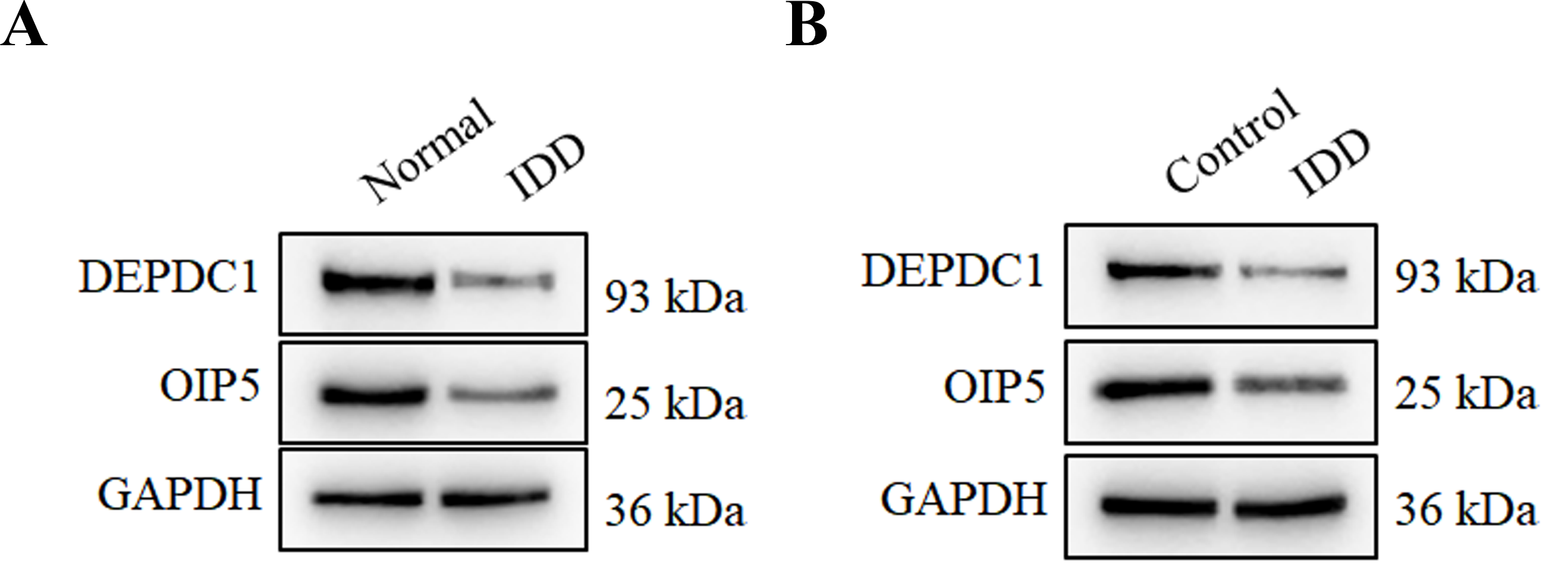
**

**Figure S3** The protein expression of two hub-genes in tissues and cells of IDD patients. **(A)** The protein expression of OIP5 and DEPDC1 in IDD and normal tissue. **(B)** The protein expression of OIP5 and DEPDC1 in NP cells in IDD and control.


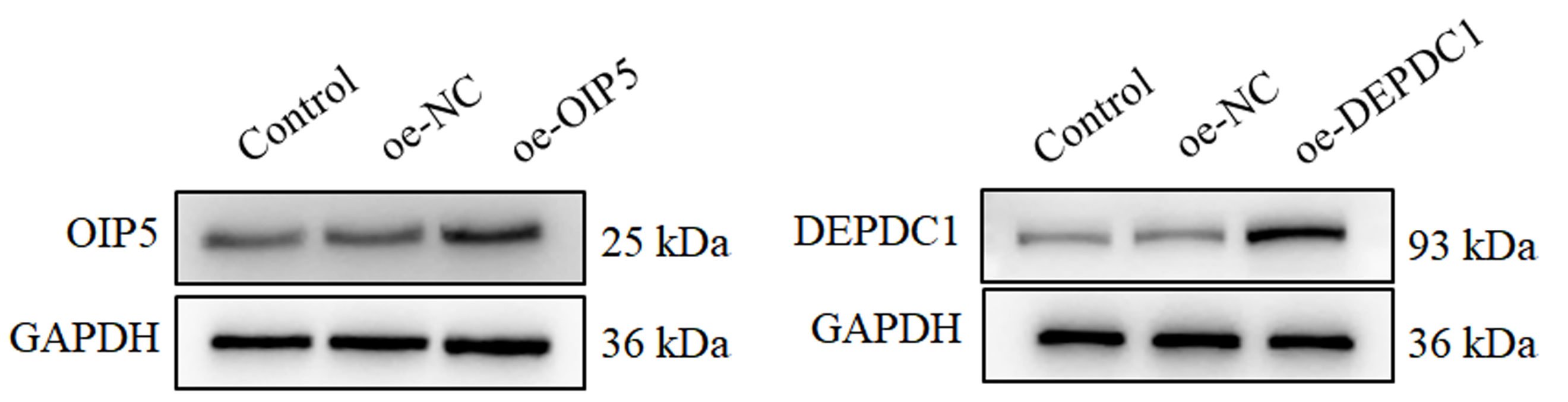


**Figure S4** Western blot was used to measure the overexpression efficiency of OIP5 and DEPDC1 in NP cells.
